# Laser-assisted fabrication of gold nanoparticle-composed structures embedded in borosilicate glass

**DOI:** 10.3762/bjnano.8.244

**Published:** 2017-11-21

**Authors:** Nikolay Nedyalkov, Mihaela Koleva, Nadya Stankova, Rosen Nikov, Mitsuhiro Terakawa, Yasutaka Nakajima, Lyubomir Aleksandrov, Reni Iordanova

**Affiliations:** 1 Institute of Electronics, Bulgarian Academy of Sciences, 72 Tsarigradsko Chaussee blvd., 1784 Sofia, Bulgaria; 2 Department of Electronics and Electrical Engineering, Keio University, 3-14-1 Hiyoshi, Kohoku-ku, Yokohama 223-8522, Japan,; 3 Institute of General and Inorganic Chemistry, Bulgarian Academy of Sciences, bld. 11, Acad. Georgi Bonchev str. 1113 Sofia, Bulgaria

**Keywords:** 2D and 3D nanoparticle fabrication, gold nanoparticles in glass, laser nanostructuring, optical properties of composite materials

## Abstract

We present results on laser-assisted formation of two- and three-dimensional structures comprised of gold nanoparticles in glass. The sample material was gold-ion-doped borosilicate glass prepared by conventional melt quenching. The nanoparticle growth technique consisted of two steps – laser-induced defect formation and annealing. The first step was realized by irradiating the glass by nanosecond and femtosecond laser pulses over a wide range of fluences and number of applied pulses. The irradiation by nanosecond laser pulses (emitted by a Nd:YAG laser system) induced defect formation, expressed by brown coloration of the glass sample, only at a wavelength of 266 nm. At 355, 532 and 1064 nm, no coloration of the sample was observed. The femtosecond laser irradiation at 800 nm also induced defects, again observed as brown coloration. The absorbance spectra indicated that this coloration was related to the formation of oxygen deficiency defects. After annealing, the color of the irradiated areas changed to pink, with a corresponding well-defined peak in the absorbance spectrum. We relate this effect to the formation of gold nanoparticles with optical properties defined by plasmon excitation. Their presence was confirmed by high-resolution TEM analysis. No nanoparticle formation was observed in the samples irradiated by nanosecond pulses at 355, 532 and 1064 nm. The optical properties of the irradiated areas were found to depend on the laser processing parameters; these properties were studied based on Mie theory, which was also used to correlate the experimental optical spectra and the characteristics of the nanoparticles formed. We also discuss the influence of the processing conditions on the characteristics of the particles formed and the mechanism of their formation and demonstrate the fabrication of structures composed of nanoparticles inside the glass sample. This technique can be used for the preparation of 3D nanoparticle systems embedded in transparent materials with potential applications in the design of new optical components, such as metamaterials and in plasmonics.

## Introduction

The unique optical properties of noble-metal nanoparticles continue to attract the attention of researchers, especially in view of the potential industrial applications. The basic characteristics of the interaction of an electromagnetic field with these objects are defined by the efficiency of the plasmon excitation (i.e., collective oscillations of the free electrons at a specific frequency defined by the properties of the metal and the surrounding medium) [1]. On resonance, when the electromagnetic field frequency equals that of the plasmon, the optical properties of the nanoparticles drastically change. The extinction coefficient is strongly enhanced, and its value may exceed the off-resonance extinction coefficient value by orders of magnitude [2].

While at the turn of the century research interest was mainly directed at studying and describing the properties of single nanoparticles and their two-dimensional ensembles, in recent years, research has tended to deal with more complex systems, including three-dimensional architectures, composite materials consisting of nanoparticles, and three-dimensional ordered nanoparticle ensembles. The studies conducted [3–6] indicated that the optical properties of such systems are governed by additional parameters, namely, the interparticle distance, and the geometry of the ensemble and its orientation with respect to the electromagnetic field polarization. These complex interactions are yet to be described in detailed theory; thus, the optical properties of such systems requires further study. An example of the specific properties of such ensembles is the so-called “plasmon coupling”, an effect that takes place between closely located nanoparticles [7], whose impact on the optical properties of the nanoparticle systems is not fully described. This effect is also responsible for the specific distribution of the electromagnetic field in the near-field zone, characterized by “hot spot” areas with a strongly enhanced intensity [2].

The practical applications of the optical properties of complex nanoparticle systems usually require a “carrier medium”, i.e., a dielectric matrix transparent in the spectral range of the metal structure’s plasmon resonance. The resulting composite material from the research discussed in this work shows optical properties similar to those of both systems – transparent over a wide spectral range with specific features defined by the characteristics of the metal nanoparticles. The presence of nanoparticles may also lead to a significant change in the material’s refractive index, where its value and sign depend on the particle concentration [8]. The studies performed on the optical properties of such composites demonstrated that, in addition to these sum effects, these materials may express qualitative changes in their response in the process of the interaction with an electromagnetic field. It was shown that embedding noble metal nanoparticles into dielectric oxide and polymer matrices results in a large increase of the third-order nonlinear optical susceptibility, an enhancement of the photoluminescence signal from the matrix, a metamaterial-like behavior, and the possibility of modifying the conductivity of the semiconductor by varying the particle concentration [9–11]. Any further progress regarding detailed studies of or in finding novel applications for these materials is still hampered by the lack of efficient fabrication techniques. Although various techniques, such as chemical reactions, ion implantation, ion exchange, and co-deposition [12–14] have been developed, major requirements for production efficiency, including reducing production time and cost, have yet to be met. The existing technologies cannot provide efficient control and reproducibility of the spatial characteristics of complex 3D nanoparticle ensembles. The practical implementation of methods that can avoid these drawbacks would certainly have a strong impact on the detailed study of the specific properties of such systems and on the development of new applications.

A technique has been demonstrated [15–22] based on the interaction of laser radiation with noble-metal-doped simple oxides, glasses and polymers, that is capable of forming and manipulating nanoparticles with a high spatial selectivity. Thereby, ion-doped (gold, silver, copper) materials that are transparent in the visible wavelength range (e.g., silicate, phosphate and borate glasses, titanium and aluminum oxides, and polymers) are processed by laser radiation with appropriate parameters. Metal nanoparticles are formed within the irradiated area after a subsequent thermal annealing. This photoreduction process has been achieved by using CW lasers oscillating in the UV [18] but more often by femtosecond lasers [15–17,22]. Nanosecond laser irradiation has rarely been reported as a source of metal reduction in glasses [23]. The studies cited indicated that the performance, i.e., the optimal process conditions, depends strongly on the glass type and composition. Due to the complexity of the process involved, it is difficult to devise a clear and detailed picture of the underlying physical phenomena and of their interplay. These include the mechanisms of laser irradiation interaction with and absorption by these materials, the development and evolution of defects and their role in the formation of nanoparticles, and the influence of the processing parameters. It is necessary to understand and adequately describe the relation between the initial material composition, the laser parameters and the annealing conditions, as well as the characteristics of the formed nanoparticles and their complex ensembles. All of this hampers the development of the method and its applications.

In this paper, we describe laser-induced spatially selective preparation of nanoparticles in borosilicate glass. The study reveals the impact of a wide variety of processing parameters that have not been previously considered, such as the use of nanosecond laser pulses at different wavelengths, and highlights characteristic dependences that may assist in the understanding and acceleration of potential applications of this unique method of producing 3D nanoparticle structures. This type of glass in combination with gold nanoparticles has not been considered in previous studies, but is of considerable interest regarding the design of high-quality optical elements.

## Experimental

The material of interest was borosilicate glass with composition (in wt %) as follows: 50% SiO_2_, 20% Al_2_O_3_, 20% B_2_O_3_, 5% CaO, 2% Li_2_O, 3% MgO, where AuCl_3_ is added to the initial mixture in an amount ensuring 0.015 wt % of Au in the final glass sample. The mixed material is melted in a Pt crucible and kept at a temperature of 1450 °C for three hours. After cooling, the glass sample is cut into pieces with dimensions of 10 × 10 × 3 mm and polished. We chose borosilicate glass because of its low thermal expansion coefficient and good resistance to the high thermal gradients likely to be induced by laser processing. The samples were irradiated by nanosecond and femtosecond laser pulses with different parameters and the optical response was studied. In the nanosecond pulse case, we employed a Nd:YAG laser systems (Lotis) operating at four wavelengths – 1064 nm, 532 nm, 355 nm and 266 nm, with a pulse duration of 12 ns. The laser light was focused by a fused silica lens with a focal length of 200 mm. The femtosecond processing was performed by means of a Ti:sapphire system (Libra, Coherent) emitting pulses with a duration of 150 fs and a repetition rate of 1 kHz at the wavelength of 800 nm. A lens with a focal length of 250 mm was used to focus the radiation on the sample. The optical properties of the samples were studied via their absorbance spectra (HR 4000 spectrometer, Ocean Optics) in the range 200–1000 nm. The experimental procedure included thermal annealing of the glass samples in a standard controllable oven. The glass transition temperature and the thermal stability of the glass samples prepared were determined by differential thermal analysis (DTA) (Setaram, Labsys Evo 1600) at a heating rate of 10 °C/min (±1 K) in air. The glass structure after the laser processing and thermal annealing was investigated by transmission electron microscopy (TEM) (JEOL JEM 2100). The EMCAT/EMFIT computer program [24] was used to interpret the selected-area electron diffraction (SAED) patterns and to identify the phase composition of the samples. Optical images of the processed areas were obtained using an Optica B-150 microscope.

## Results and Discussion

### Glass characteristics

**Figure 1:**
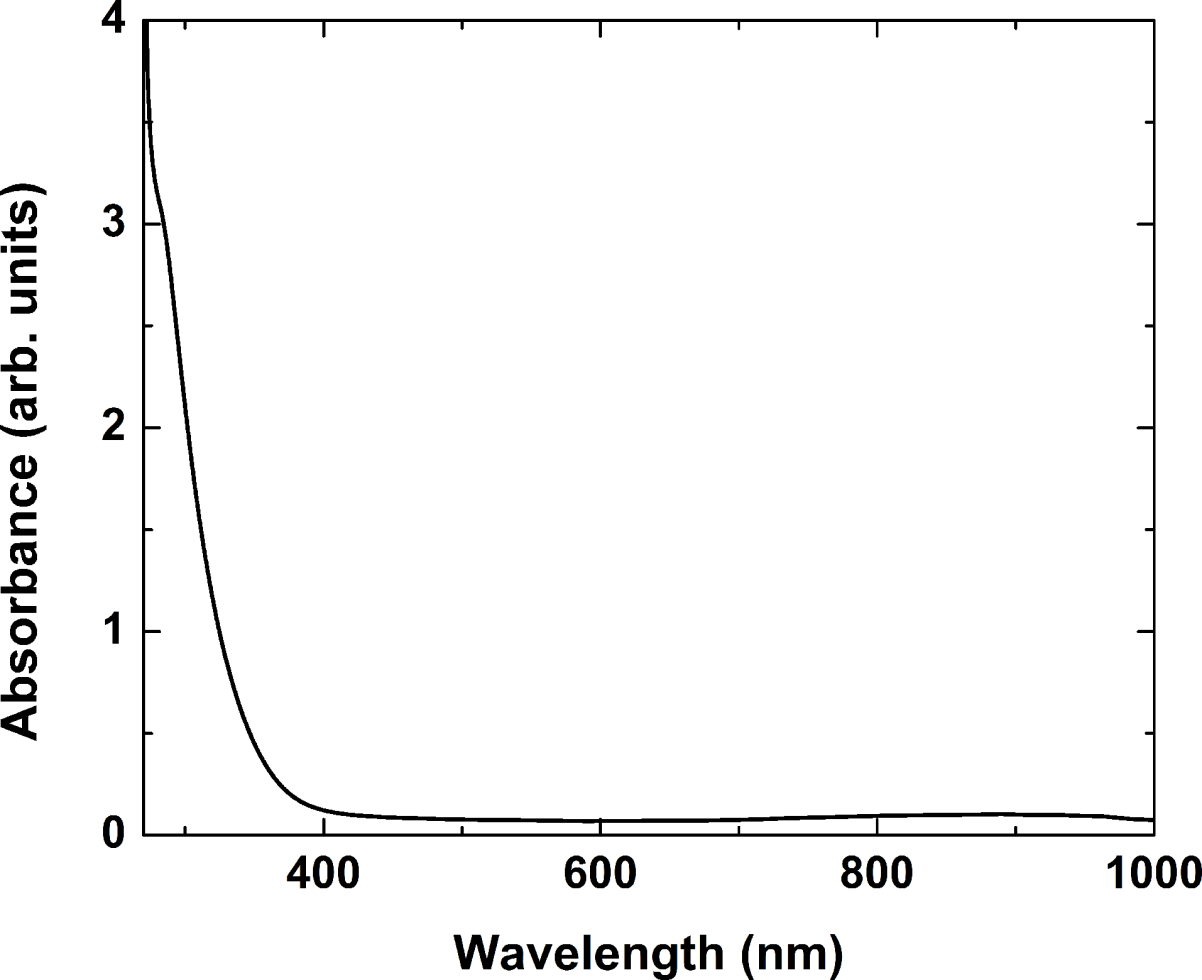
Optical absorbance spectrum of the as-prepared borosilicate glass.

The as-fabricated glass was transparent in the visible and near IR spectral regions (Figure 1). The absorbance spectrum shown was taken with respect to air. The absorbance rose sharply, with the material becoming opaque at wavelengths shorter than approximately 350 nm. The experiments on thermal annealing of the glass at different temperatures proved that its transparency was preserved up to temperatures of about 620 °C. At 650 °C, the sample acquired a clear pink color, which became darker as the temperature was raised.

**Figure 2:**
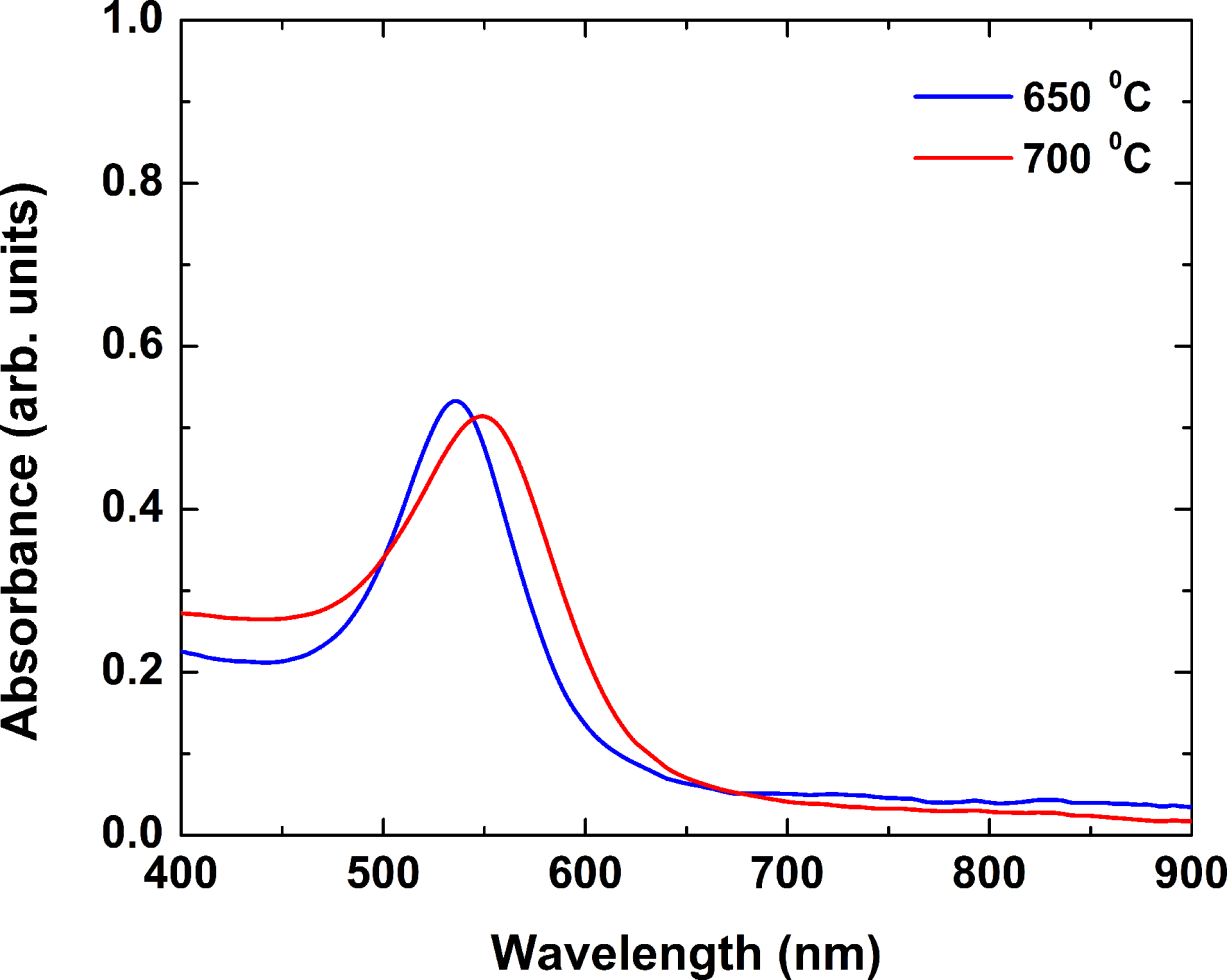
Absorbance spectra of borosilicate glass samples annealed at 650 °C (blue curve) and at 700 °C (red curve) for 30 min.

Figure 2 presents the absorbance spectra of samples annealed for 30 min at 650 °C (blue curve) and 700 °C (red curve). The spectra exhibited a well-pronounced peak at 535 nm for the sample annealed at 650 °C and at 550 nm for the sample annealed at 700 °C.

**Figure 3:**
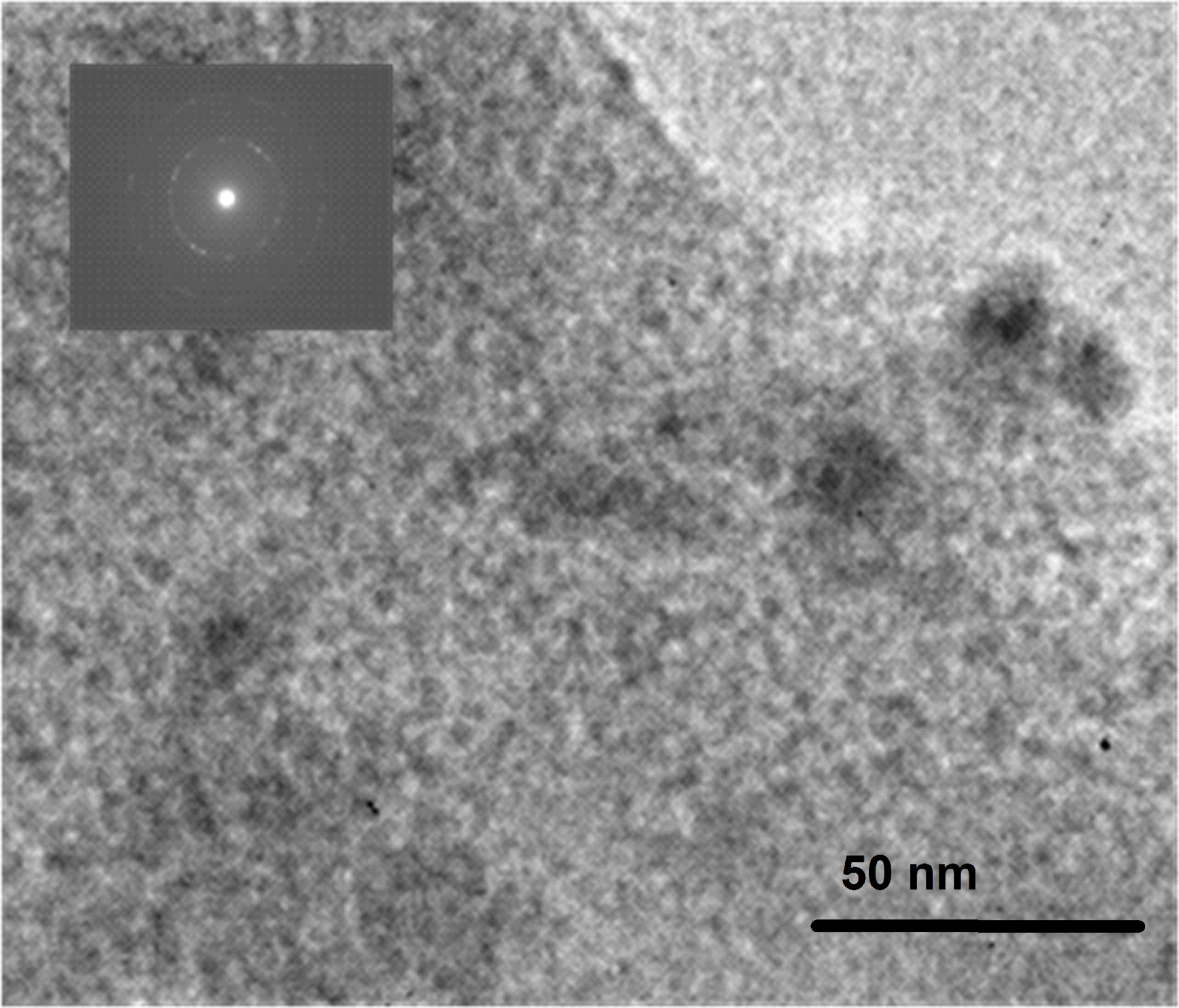
TEM image of the glass sample annealed at 700 °C. The inset shows an SEAD image taken from one of the darker spots in the image.

To clarify the origin of the coloring observed, we used high-resolution TEM. Figure 3 is an image of the structure of the sample annealed at 700 °C showing the presence of darker zones. The inset is an SEAD image taken from one of these areas; it indicated the existence of a polycrystalline structured component, while the analysis of the diffraction pattern revealed that the material was metallic gold. The high-resolution TEM image pointed to cubic phase Au, space group Fm-3m (PDF 65-2870). Thus, it can be assumed that annealing the glass samples at temperatures above approximately 620 °C resulted in the formation of gold nanoparticles inside the sample.

The appearance of gold nanoparticles is attributed to the thermally induced reduction of gold ions and the growth of nanoparticles [25]. The analysis of gold-containing glass samples reported earlier [26] showed that in the as-fabricated, transparent samples the gold is monovalent. The temperature increase during the annealing process results in the reduction of Au ions to Au atoms, the latter subsequently coalescing to form nanoparticles. The DTA performed showed that the glass transition temperature is 603 °C. Above this temperature, the atom mobility increases significantly, facilitating the growth of clusters. A further increase of the temperature (or annealing time) results in a higher efficiency of these processes, and respectively, leads to the development of bigger particles. The optical properties of gold nanoparticles in the visible spectral range are determined by the efficient plasmon excitation. The value of the plasmon wavelength is related to the particle characteristics, the interparticle distance and the properties of the environment [1]. For particles with a diameter of up to a few tens of nanometers that are dispersed in glass, the corresponding wavelength is in the range 520–530 nm [27]. With the increase of the particle size, one observes a red shift of the plasmon resonance wavelength. Therefore, the change of the glass color upon heating can be explained by the appearance of Au nanoparticles and their increasing size, i.e., the shift of the plasmon resonance wavelength position, as seen in Figure 2.

### Nanosecond-pulse processing

**Figure 4:**
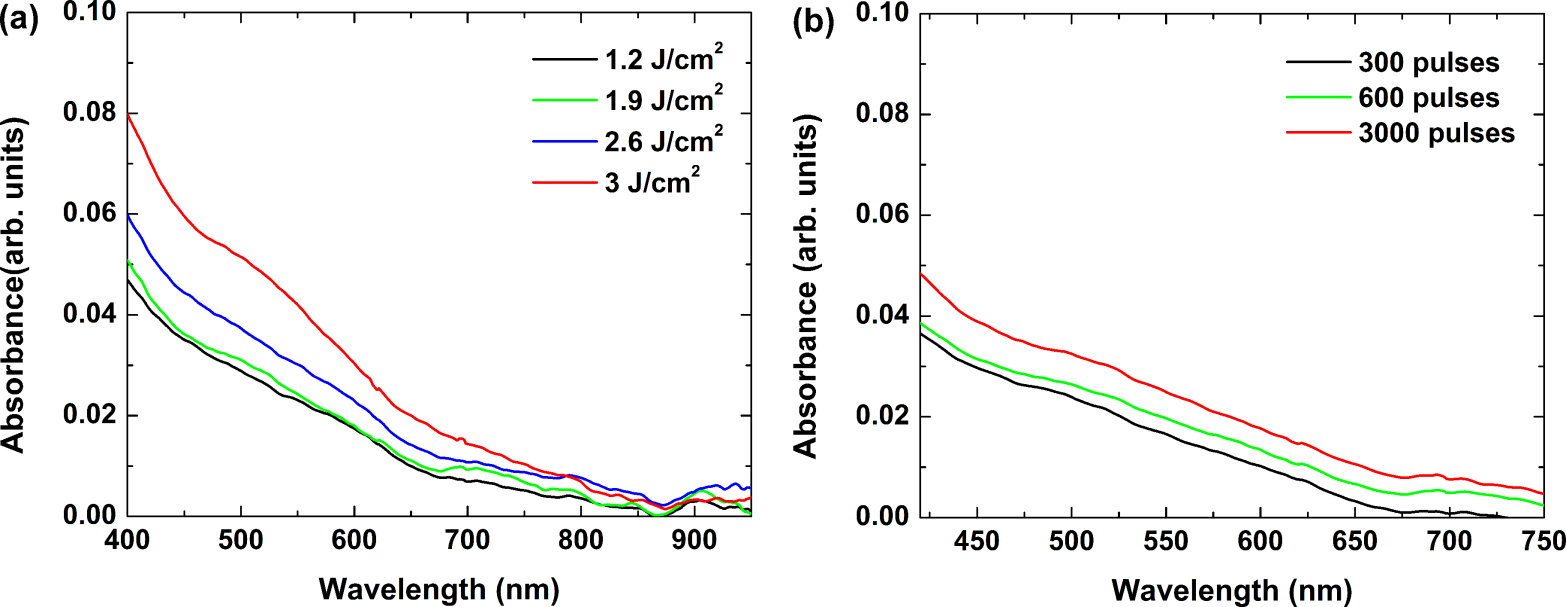
Absorbance spectra of glass samples after irradiation by nanosecond laser pulses at 266 nm. a) Varying laser fluence with pulse number fixed at 600, and b) varying pulse number at a fixed laser fluence of 1.9 J/cm^2^.

To determine the conditions for formation of nanoparticles in glass samples, we irradiated the samples with four wavelengths emitted by the Nd:YAG nanosecond laser. For each wavelength, a different laser fluence and number of pulses were used. In the 266 nm wavelength case, a clear change of the glass color was visible to the naked eye, namely, the irradiated areas acquired a brown coloration. Figure 4 shows the absorbance spectra of the areas in the glass sample irradiated by different laser fluences with the pulse number fixed at 600 (Figure 4a), and when varying the pulse number at a fixed laser fluence of 1.9 J/cm^2^ (Figure 4b). The general trend observed in these spectra is that the absorbance decreases as the wavelength increases; above 700 nm, it approaches that of the non-irradiated glass. Two specific regions can be distinguished in all cases – an increase of the absorbance at wavelengths less than 450 nm, and a peak in the range from 470 to 650 nm. These spectral characteristics can be attributed to absorption by color centers appearing in the glass sample due to irradiation. Although the mechanism of their formation is yet to be explained, and their nature is still to be clarified, some comments can be made. The coloration observed after laser irradiation can be assigned to oxygen deficiency related to intrinsic defects in the silica matrix [16,28]. Since the photon energy at 226 nm is higher than the Si–O bonding energy (4.5 eV), the laser radiation may cause direct bond breaking. The absorption below 400 nm is related to two types of defects: an unpaired electron in the sp^3^ orbital of a single silicon atom with an oxygen vacancy, and a hole trapped in an oxygen vacancy. The defects that absorb in the visible spectral range are related to nonbridging oxygen hole centers. Since the glass samples used in this study contain large amounts of B_2_O_3_ and Al_2_O_3_, a contribution of defect absorption induced by modification of the bond in these oxides should also be considered. According to Sakka [29], defects induced in borate glasses have strong absorption in the visible range, peaking at about 590 nm. For alumina-based glasses, the main absorption bands related to defects are in the UV spectral range [30].

As seen in Figure 4, increasing the laser fluence and the pulse number applied does not affect the absorption band positions, but rather their intensity. This implies that these two parameters contribute to the density of the induced defects only. At a fluence of greater than 3 J/cm^2^, ablation of the sample was observed. It should be mentioned that the absorption of the laser energy at 266 nm occurs within a thin surface layer due to the high absorbance of the material in this range (see Figure 1). This results in an increase of the absorbance of the sample due to defect formation in the visible spectral range of only a few percent, as can be seen in Figure 4.

Irradiation at wavelengths of 355, 532 and 1064 nm did not cause any observable coloration of the glass samples treated. This result was obtained for a wide variation of the laser fluence, up to the ablation threshold, and for a different number of laser pulses up to 9000.

**Figure 5:**
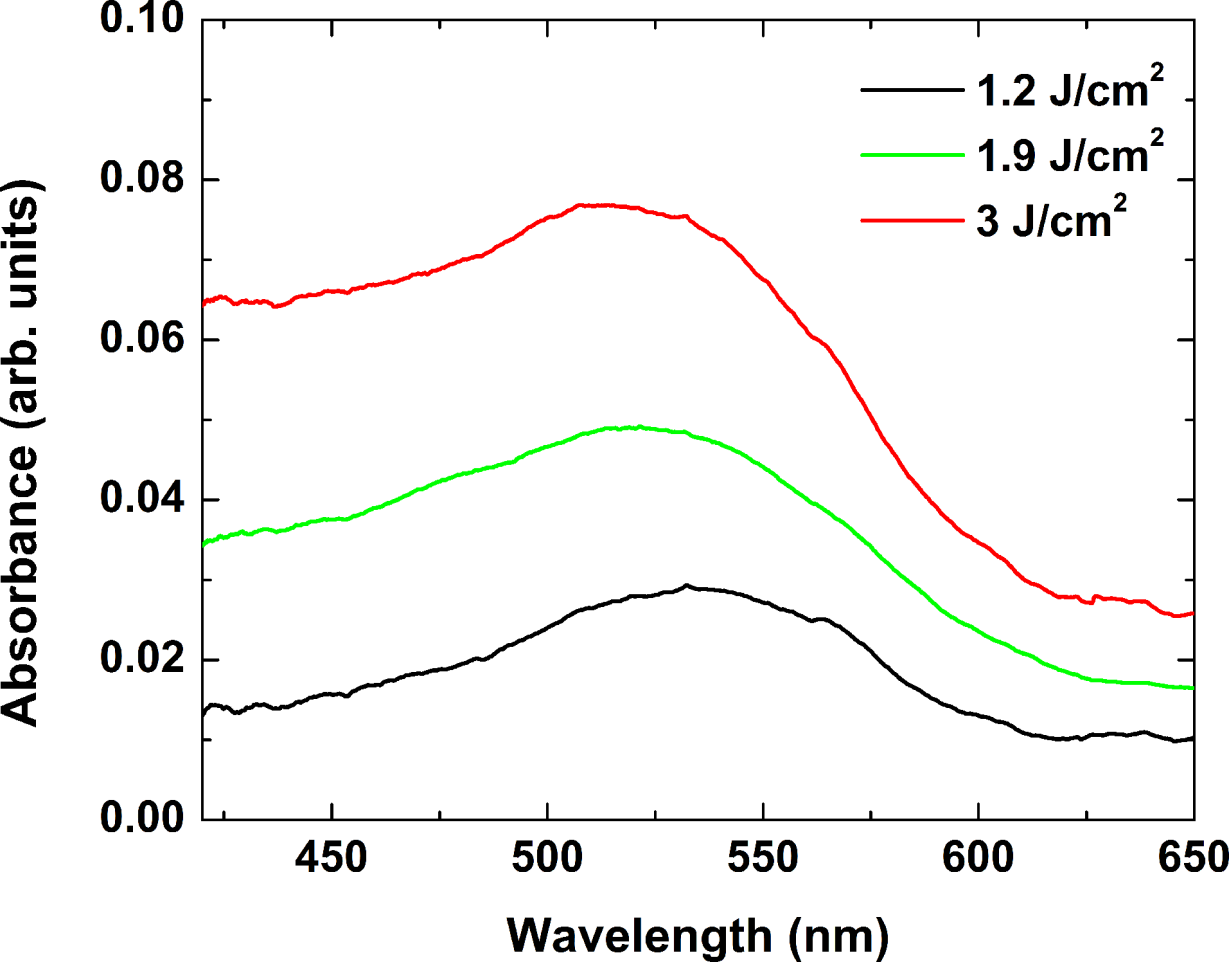
Absorbance spectra of samples irradiated by 600 ns laser pulses at a wavelength of 266 nm with different laser fluences and subsequently annealed at 600 °C for 30 min.

After laser irradiation, the glass samples were annealed for 30 min at 600 °C. These conditions were found to be optimal regarding the material response. At higher temperatures or longer annealing times, the entire sample became pink colored. At temperatures below 500 °C, the coloration induced by laser irradiation at 266 nm disappeared and the glass became again transparent in the laser-irradiated areas. This effect has to do with the thermally induced relaxation of the color centers [31]. Annealing the sample irradiated at 266 nm at 600 °C produced pink coloration in the areas of laser irradiation. Figure 5 presents the absorbance spectra of the annealed samples irradiated at different laser fluences with the number of laser pulses fixed at 600. The spectra of the sample following laser irradiation only are shown in Figure 4a. Obviously, annealing cancels out the rise in the absorbance at wavelengths shorter than 450 nm. In the visible range, we observed a well-expressed peak. The position of this peak depended on the laser fluence, namely, for a fluence of 1.2 J/cm^2^ it was at 532 nm and shifted to 520 nm when the laser fluence of 3 J/cm^2^ was applied.

Annealing the samples irradiated at 355, 532and 1064 nm at fluences below the ablation threshold did not affect their absorbance spectra under all irradiation conditions tested. Therefore, the appearance of color centers in the glass is a necessary condition for the formation of nanoparticles at the annealing stage. It should be mentioned, however, that weak pink coloration was seen after annealing the samples where laser irradiation had caused ablation at the above-mentioned wavelengths. This phenomenon requires further study.

### Femtosecond-pulse processing

**Figure 6:**
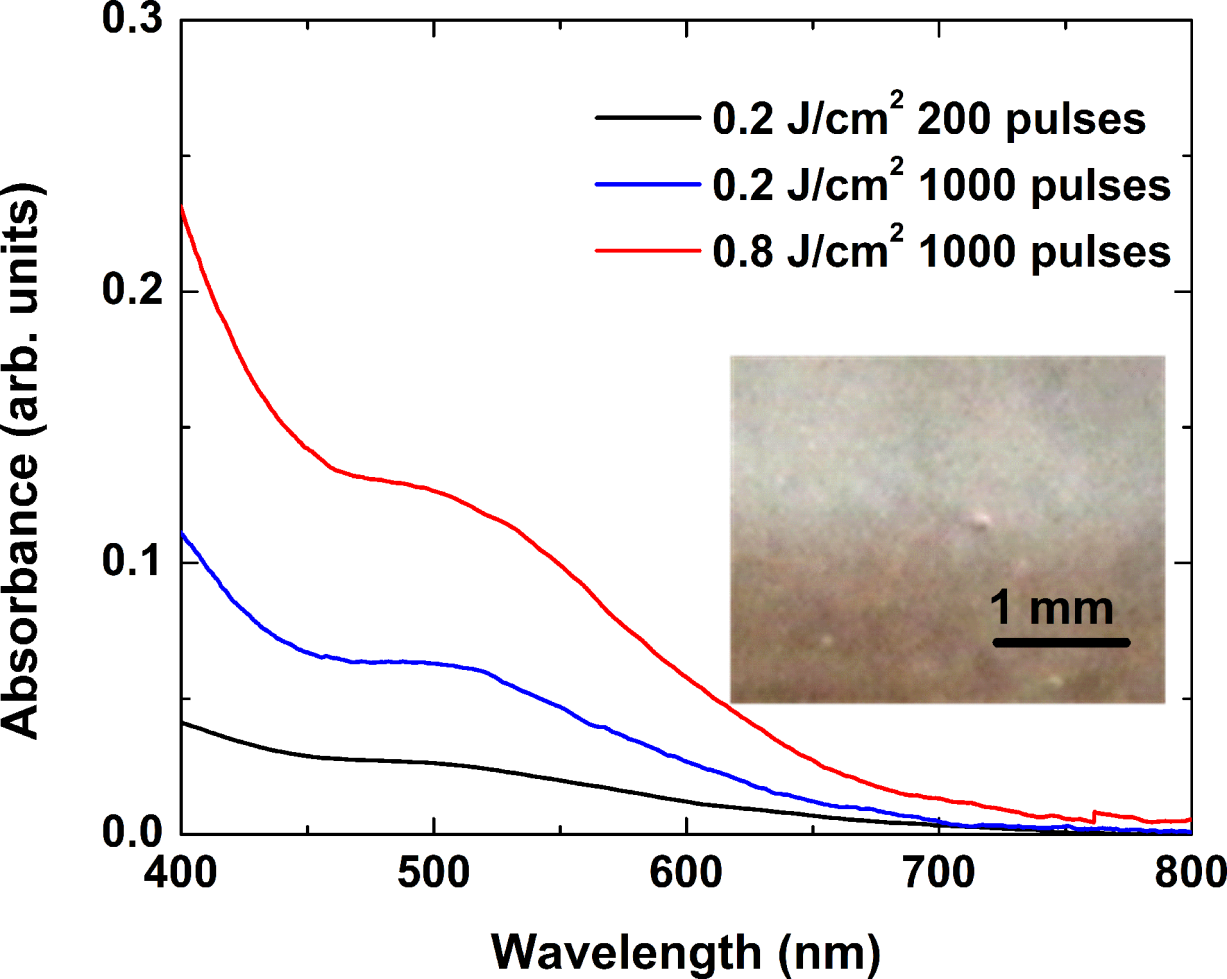
Absorbance spectra of glass samples irradiated by femtosecond laser pulses at 0.2 J/cm^2^, 200 pulses (black), 0.2 J/cm^2^, 1000 pulses (blue), and 0.8 J/cm^2^, 1000 pulses (red). The inset shows an optical microscope image of the edge of the irradiated area at a fluence of 0.8 J/cm^2^ after 1000 laser pulses.

Laser irradiation of the glass samples by femtosecond laser pulses at 800 nm gave rise to distinct coloration of the processed zones. The effect was visible to the naked eye using a wide range of fluences, starting from about 0.2 J/cm^2^ up to the ablation threshold of about 2.6 J/cm^2^. When ablation of the sample occurred, no coloring was observed in the vicinity of the ablated area. Optical microscopy inspection showed that once the glass became colored, it spanned the entire sample thickness. Figure 6 shows absorbance spectra of glass samples irradiated by femtosecond laser pulses at 0.2 J/cm^2^, 200 pulses (black line); 0.2 J/cm^2^, 1000 pulses (blue line); and 0.8 J/cm^2^, 1000 pulses (red line). The inset shows an image of the edge of the area irradiated at a fluence of 0.8 J/cm^2^ with 1000 laser pulses. As one can see, increasing both the laser fluence and the number of pulses increases the absorbance. As in the case of irradiation using nanosecond pulses at 266 nm, two bands with similar characteristics can be identified. Thus, one can conclude that femtosecond radiation induces the same type of defects in the glass matrix.

**Figure 7:**
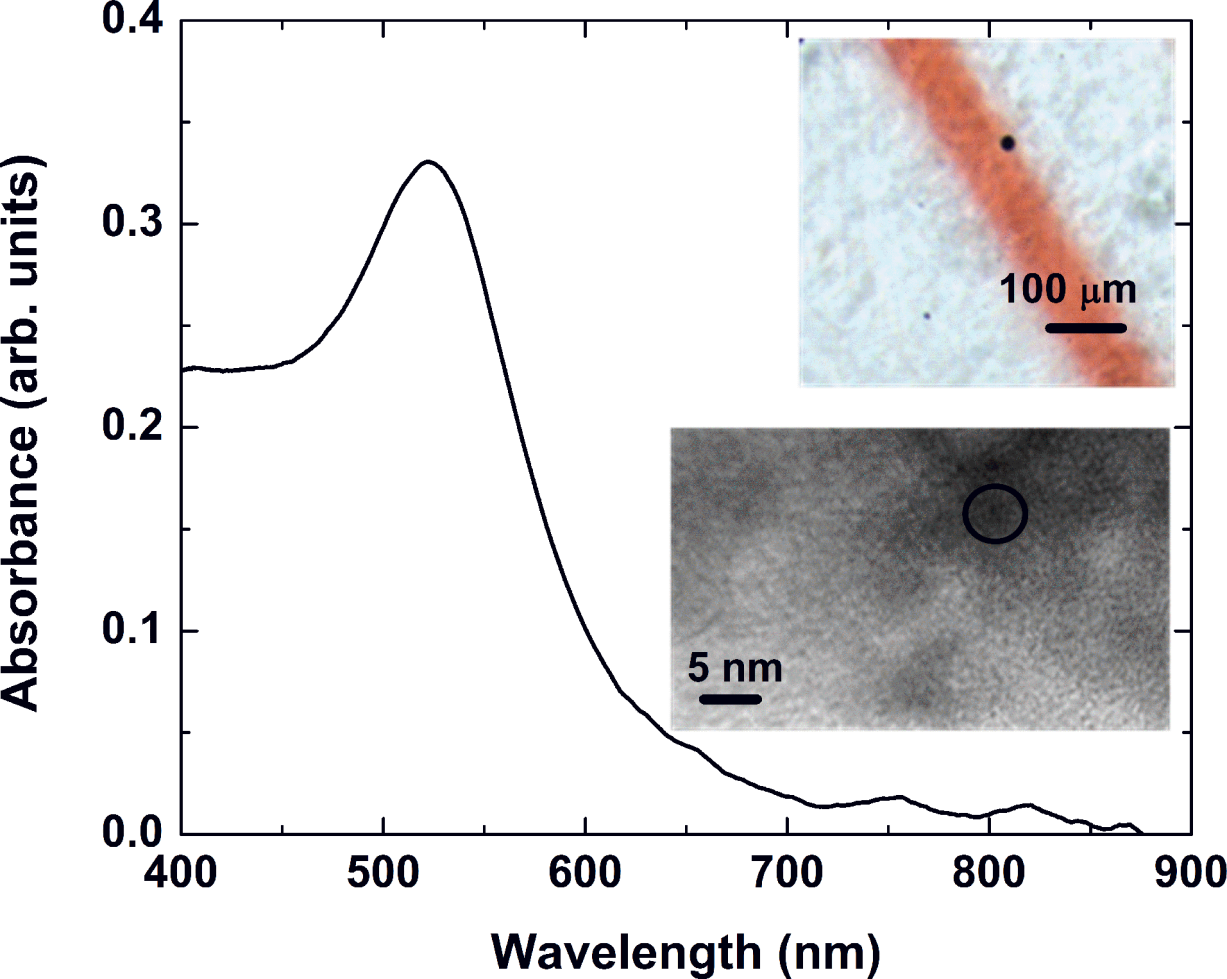
Absorbance spectrum of a laser-irradiated zone whose optical microscope image is presented in the inset (top right). The zone was obtained by moving the sample at a speed ensuring the overlap of 1000 femtosecond pulses at a fluence of 0.8 J/cm^2^. The high-resolution TEM image shows the structure of the material in the modified zone (bottom right). The zone marked by a circle indicates an area where the cubic structure of gold could be detected.

The samples irradiated by femtosecond pulses were annealed under the same conditions as in the case of nanosecond laser processing. Again, the thermal treatment produced a red coloring of the irradiated areas. Figure 7 shows the absorbance spectrum of a modified zone, whose optical microscope image is presented in the inset. The colored line was formed by moving the sample at a speed ensuring the overlap of 1000 pulses. The laser fluence was 0.8 J/cm^2^. The spectrum shows a well-defined peak centered at 525 nm. The high-resolution TEM image of the red-colored glass revealed the presence of gold nanoparticles (the inset in Figure 7, bottom right). In the inset of Figure 7, gold was detected in the area marked by a black circle. Due to the low concentration of particles in the TEM probe, reliable size-distribution data could not be obtained; a rather rough estimate yielded a particle size of about 5 nm. Thus, we believe that the absorbance peak is related to absorption by gold nanoparticles capable of surface plasmon resonance. The TEM image also shows crystal phases that are not recognized as gold (the dark areas surrounding the black circle). One can speculate that local crystallization of the glass takes place after the treatment; further analysis is needed to interpret these effects.

**Figure 8:**
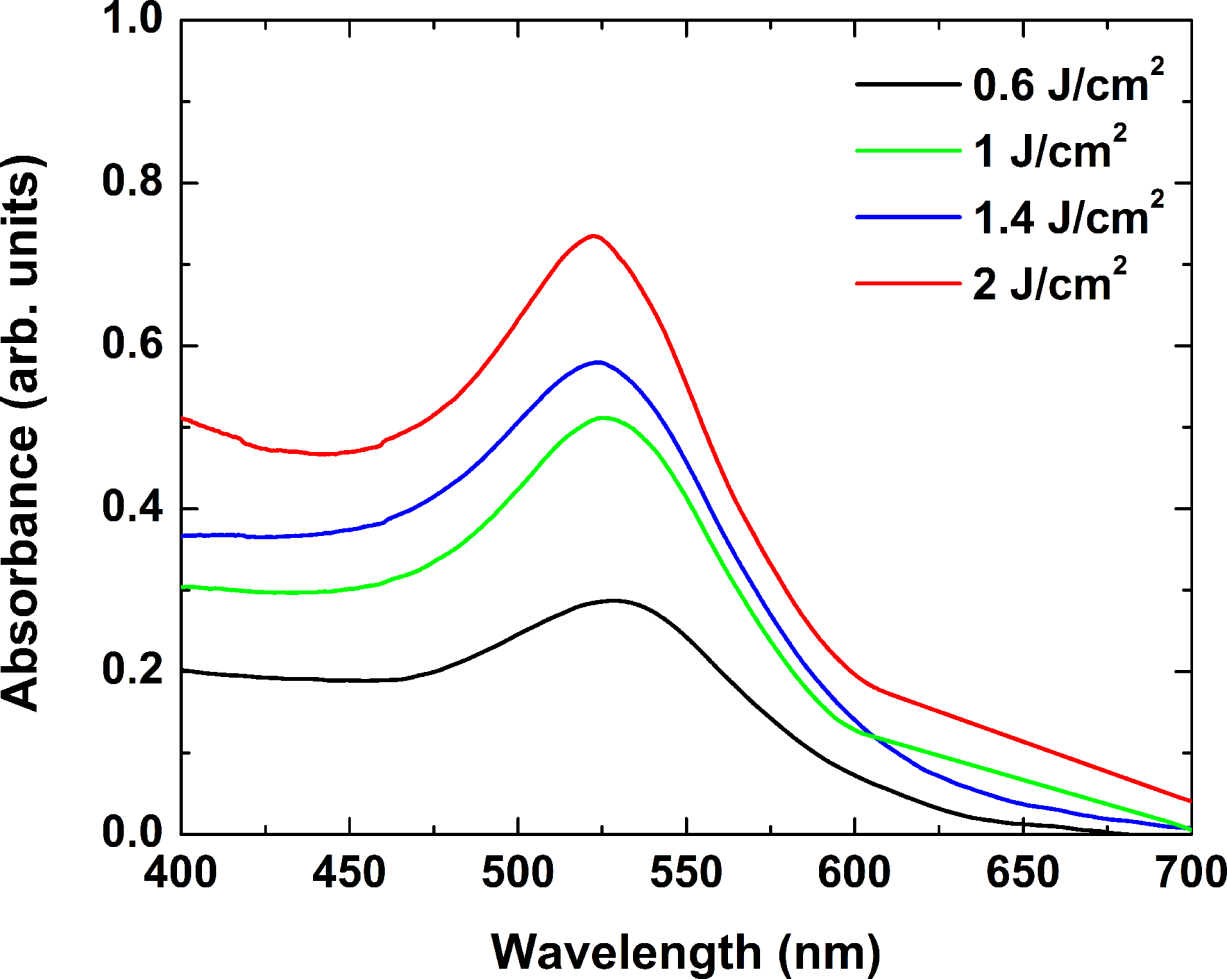
Absorbance spectra of glass samples irradiated by 10,000 pulses with laser fluences of 0.6 J/cm^2^ (black line), 1 J/cm^2^ (green line), 1.4 J/cm^2^ (blue line), and 2 J/cm^2^ (red line). The samples were then annealed at 600 °C for 30 min.

The laser-processing parameters were found to influence the position and the height of the absorbance peak. Figure 8 shows the absorbance spectra of glass samples irradiated by 10,000 pulses with laser fluences of 0.6 J/cm^2^ (black line), 1 J/cm^2^ (green line), 1.4 J/cm^2^ (blue line) and 2 J/cm^2^ (red line), followed by annealing at 600 °C for 30 min. Increasing the laser fluence led to an increase of the absorbance, while the peak position shifted from 528 nm at a fluence of 0.6 J/cm^2^ to 522 nm at 2 J/cm^2^. A similar peak-shifting effect was observed when the number of laser pulses applied was increased from 100 to 10,000 at a fixed laser fluence of 1 J/cm^2^.

In an attempt to understand the absorbance spectra behavior as a function of the processing parameters, we conducted a simulation of the optical properties of gold nanoparticles in glass. Since the experimental spectra showed no presence of multimode plasmon excitation, and bearing in mind the low initial concentration of gold in the glass, the simulation assumed that the nanoparticles formed were spherical, well-separated in a homogeneous glass environment, and any influence of the scattered field on their optical response could be neglected. The value of 1.53 was used for the refractive index, as determined by the ellipsometric analysis of the glass. The simulation was based on Mie’s theory, which yields for the extinction cross section [32]:

[1]
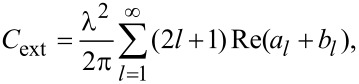


where

[2]
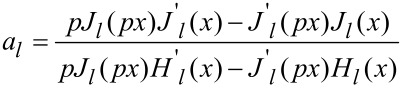


[3]
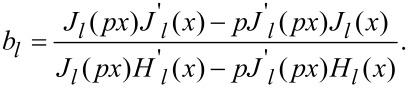


In these equations *p* = (ε/ε_m_)^1/2^, *x* = 2π*R*/λ, *J_l_* and *H_l_* are the Riccati–Bessel functions, ε and ε_m_ are the dielectric functions of gold and the matrix material (glass), *R* is the particle radius, and λ is the electromagnetic field wavelength. The dielectric function of glass is assumed constant in the visible spectral range. Since the TEM images indicated that the nanoparticle diameter under the conditions described was in the range of few nanometers, the dielectric function of gold should include a size correction. This is necessary due to the fact that the electron-free path (about 40 nm for Au) is larger than the particle diameter [32]. The influence of the particle size can be taken into account by a damping parameter modification [33]

[4]
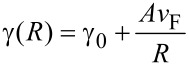


included in the expression of the dielectric function. Here γ is the damping parameter, and ν_F_ is the Fermi velocity. The values for γ_0_, and *A* are taken from [33]. The dielectric function and the corresponding parameters give an adequate description of the optical properties of gold over a wide spectral range according to [34]. Since the extinction spectrum of small nanoparticles is dominated by their absorption [32], it is used in the analysis presented below.

The reliability of the model was checked by comparing the spectra of commercial colloid containing gold nanoparticles with a diameter of 10 nm (BBI International) with the simulated one. Good agreement in the position and width of the resonance band was found, which indicated the correctness of the simulation for analysis of the optical properties of the nanoparticles.

**Figure 9:**
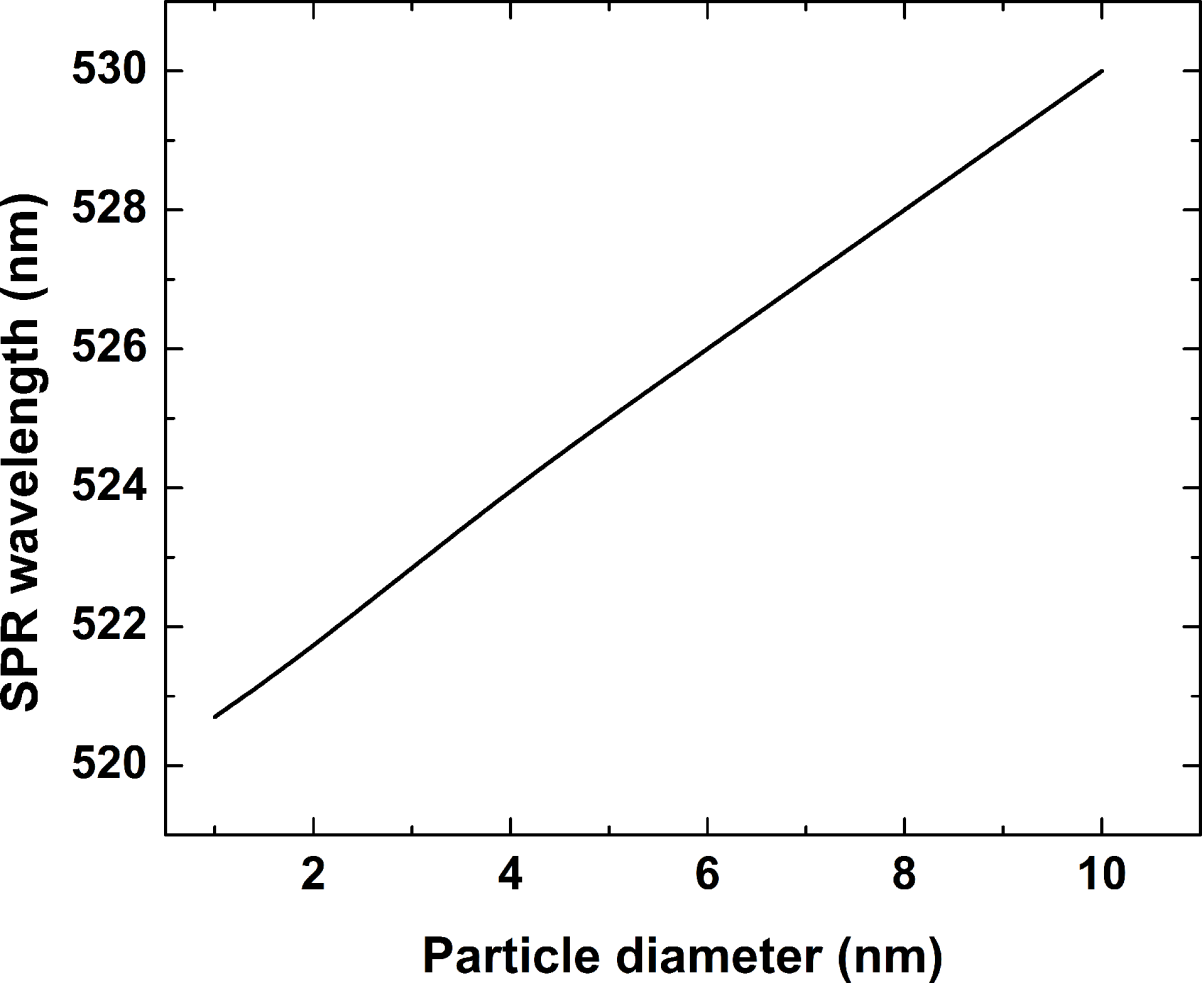
Dependence of the position of the plasmon resonance extinction band maximum on the gold nanoparticle diameter obtained from Equation 1.

Figure 9 presents the simulated dependence of the position of the plasmon resonance extinction band maximum as a function of the nanoparticle diameter. The dependence is linear, as the resonance wavelength shifts from 520 nm for a particle size of 1 nm to 530 nm for a 10-nm particle. The change of the plasmon band position experimentally observed when the laser fluence and the number of laser pulses applied are varied can, therefore, be explained by the formation of nanoparticles of different size. A comparison with the position of the resonances obtained experimentally, shown in Figure 8, points to a nanoparticle size range of 3 to 10 nm at the fluences quoted.

Femtosecond laser pulses applied with intensity near 10^13^ W/cm^2^, as used in this work, undergo multiphoton absorption in the volume of the material. This produces defects and the specific sample coloration, as shown in Figure 6. Increasing the laser fluence or the number of the laser pulses increases the defect volume density. Since the formation of nanoparticles was only observed after annealing in the irradiated areas, one could assume that these defects play a key role in nanoparticle nucleation. It is accepted [16–17] that the electrons released in the defect zones contribute to a reduction of the gold ions existing in the glass. The increase of the defect density is accompanied by more reduced ions. In the annealing process, this leads to formation of a larger number of nanoparticles of smaller size. Indeed, the increase of the absorbance at wavelengths lower than 450 nm with the increase of the laser fluence, as shown in Figure 8, implies an increase in the amount of metallic gold [35], while the blue shift of the resonance can be related to a decrease in the particle size. Note that under these conditions, the defect formation and the red-colored area expand over the total length of the sample. The absorbance spectra of the irradiated areas illustrate a change in the intensity and a shift of the resonance peak position. Under the processing parameters used in this work, this effect would not be explained by an increase in the defect-containing volume, but rather by a change of the characteristics of the particles formed during the annealing stage. It should further be noted that under all experimental conditions where glass coloring occurred, white light emission was observed accompanying the femtosecond laser irradiation. This emission is evidence of nonlinear effects taking place, as the light consisted of multiple components in the visible or even the UV spectrum. Thus, when considering the process of energy transfer from the laser pulse to the glass sample, one should also take into account more complex propagation and absorption mechanisms, whose detailed description is yet to be performed.

The similar behavior exhibited by the glass samples after irradiation by 266 nm nanosecond pulses hints at the same kinetics of nanoparticle formation. An estimate of the particle size, based on the plasmon band position taken from Figure 5 and the simulation, yields diameters in the range of 1 to 10 nm for the fluence range used.

**Figure 10:**
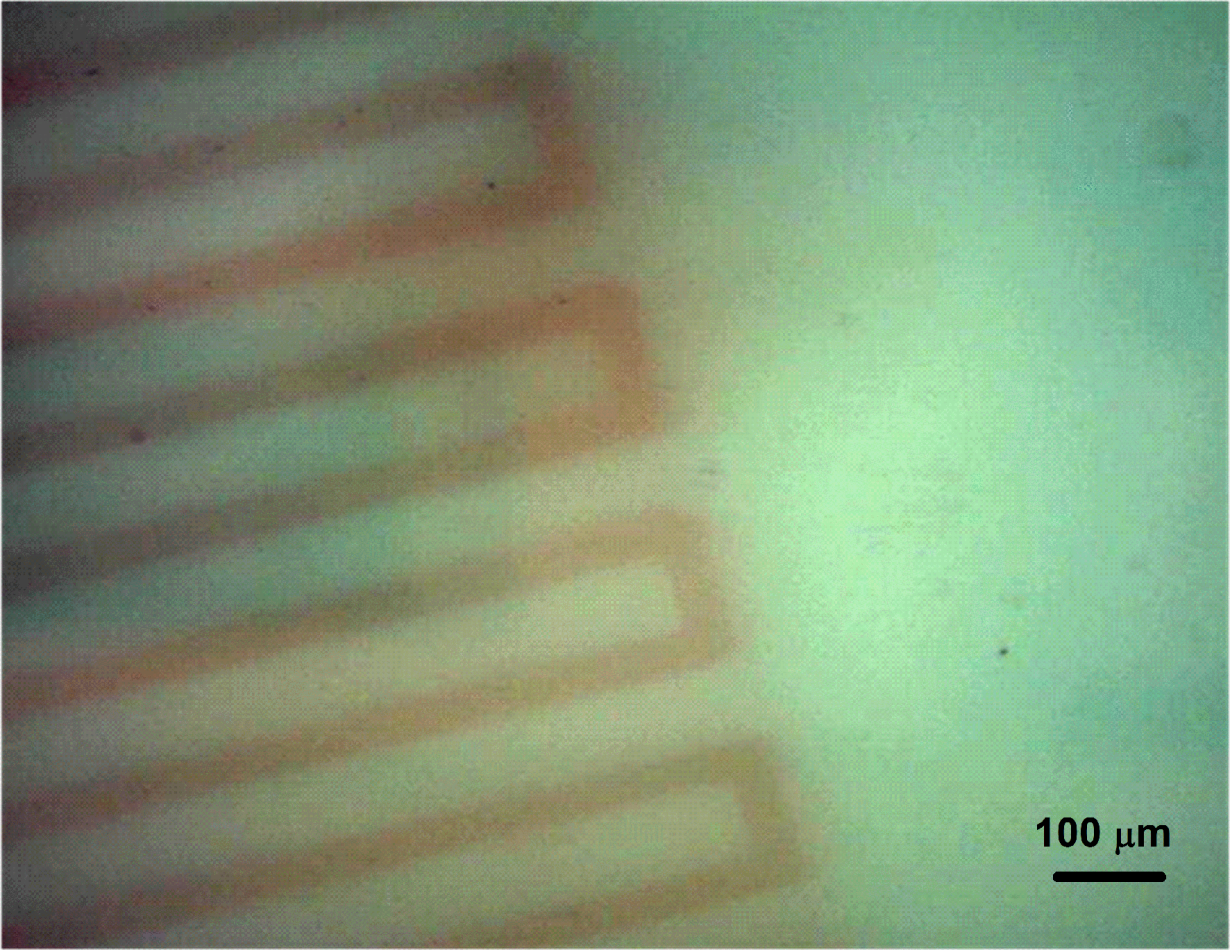
Optical microscope image of a structure fabricated 1.5 mm below the surface of a gold-doped glass sample by focusing femtosecond laser radiation using a microscope objective followed by annealing at 600 °C for 30 min. The laser fluence was 1 J/cm^2^ and the sample was moved during irradiation at a speed allowing for the overlap of 1000 laser pulses.

The fabrication method for nanoparticles presented above can be used efficiently to produce real 3D structures composed of nanoparticles. The transparency of the glass at wavelengths in the near IR spectral range allows one to generate defects inside the material by focusing the laser radiation due to the nonlinear absorption taking place when femtosecond pulses are used. After annealing, the irradiated areas will contain nanoparticles. We performed such structuring when focusing the laser radiation at a depth of about 1.5 mm below the surface of a gold-doped glass sample by means of a 40× (NA 0.65) microscope objective. Figure 10 shows an optical microscope image of the structure fabricated inside the sample. The laser fluence was 1 J/cm^2^ and the sample was moved during irradiation at a speed allowing for the overlap of 1000 laser pulses. By varying the microscope focus, the size of the structure in the *z*-direction was estimated to be about 80 μm.

Due to the high absorption of the glass in the UV range, its modification, and respectively, the areas where nanoparticles could be formed, was limited to the sample surface when nanosecond pulses at a wavelength of 266 nm were used. Thus, nanoparticle-composed structures can be formed only in a 2D manner under these conditions.

## Conclusion

A method is presented for fabrication of two- and three-dimensional structures composed of gold nanoparticles inside borosilicate glass. The nanoparticle growth process includes two steps – laser-induced defect formation and thermal annealing. Nanosecond and femtosecond laser irradiation with different parameters is used in order to induce defects into the glass. These are clearly manifested when using nanosecond pulses at a wavelength of 266 nm by a specific brown coloration of the irradiated areas. The processed areas show no morphological changes. Such a coloration is not observed for the other wavelengths emitted by the Nd:YAG laser employed. Irradiation by femtosecond laser pulses at a wavelength of 800 nm causes color changes of the irradiated areas that are spread across the entire sample thickness (3 mm) when a lens with focal length of 250 nm is used. The increased absorbance in the colored areas, which points to the formation of oxygen deficiency defects, depends on the processing conditions, namely, the absorbance increases as the laser fluence and number of applied pulses are increased. The annealing of the samples at a temperature of 600 °C for 30 min resulted in a change of the color of the irradiated areas to pink. In the case of nanosecond laser pulse processing, this is observed after irradiation at the wavelength of 266 nm only. Due to the high absorption of the glass at this wavelength, only 2D surface modifications can be implemented. The nonlinear absorption realized when femtosecond pulses are used allows one to generate defects and produce nanoparticle-containing areas in a 3D geometry. The absorbance spectra of the annealed samples display a dependence of the absorbance peak position on the laser fluence and the number of pulses applied. These features are attributed to the appearance of gold nanoparticles in the samples due to annealing, where the particle size depends on the laser-processing parameters. Based on a simulation using Mie’s theory, the diameter of the particles is estimated to be within the range of a few nanometers. The results presented can be explained by laser-induced reduction of gold ions and nanoparticle growth via diffusion of gold atoms during the annealing stage. This method offers a unique opportunity for fabrication of 2D and 3D structures composed of nanoparticles that can find application in the production of novel optical elements.
